# Severe ileum bleeding following adjuvant capecitabine chemotherapy for locally advanced colon cancer: a case report and review of the literature

**DOI:** 10.1186/s12957-021-02443-8

**Published:** 2021-11-22

**Authors:** You Zou, Shuang Liu, Jianhong Wu, Zhen Sun

**Affiliations:** 1 Department of Gastrointestinal Surgery, Tongji Hospital, Tongji Medical College in Huazhong University of Science and Technology, Wuhan, Hubei China; 2 Tongji Cancer Research Institute, Tongji Hospital, Tongji Medical College in Huazhong University of Science and Technology, Wuhan, Hubei China

**Keywords:** Bleeding, Adjuvant chemotherapy, Capecitabine, Ileitis

## Abstract

**Background:**

Capecitabine is a prodrug that is enzymatically converted to its active form, fluorouracil (also called 5-fluorouracil), which is commonly used as adjuvant chemotherapy in colorectal cancer patients. Severe gastrointestinal bleeding induced by capecitabine is rare. Here, we are presenting the first case report of surgery specimen assisted diagnosis of this uncommon condition.

**Case presentation:**

A 63-year-old Chinese male with a history of colon adenocarcinoma and right hemicolectomy presented with severe lower gastrointestinal bleeding 2 days after finishing capecitabine administration during the first cycle of XELOX adjuvant chemotherapy. Because of the negative findings of active bleeding points by digital subtraction angiography (DSA) or colonoscopy, emergency laparotomy and partial enterectomy were performed. The bloody diarrhea had resolved after surgery and a terminal ileitis was diagnosed after pathological examination of the surgical specimen.

**Conclusions:**

Terminal ileitis induced by capecitabine is likely to be underreported. It should be considered more often as a cause of severe gastrointestinal bleeding during or after treatment with capecitabine agents. Emergency surgery may achieve satisfactory outcomes if endoscopic hemostasis is ineffective.

**Highlights of this case:**

1. Gastrointestinal bleeding following capecitabine treatment in colorectal cancer patients might be life-threatening.

2. Terminal ileitis induced by capecitabine should always be considered in the differential diagnosis of severe gastrointestinal bleeding.

3. Awareness of the risk factors such as deficiency of dihydropyrimidine dehydrogenase, advanced age, or right colectomy may aid in reducing capecitabine-related morbidity.

4. When severe bleeding occurs, emergency surgery may achieve satisfactory outcomes if medical and endoscopic interventions are ineffective.

**Supplementary Information:**

The online version contains supplementary material available at 10.1186/s12957-021-02443-8.

## Background

Capecitabine is a prodrug that is enzymatically converted to its active form, fluorouracil (also called 5-fluorouracil) which is commonly used as adjuvant chemotherapy in colorectal cancer patients [1–3]. The most common gastrointestinal adverse events of capecitabine include nausea, vomiting and diarrhea [4]. However, severe gastrointestinal bleeding due to enterocolitis induced by capecitabine is rare. To date, only 11 published cases described capecitabine treatment-related ileitis, and only 1 of these cases documents bloody diarrhea after capecitabine administration [5]. However, the ileitis diagnosed in these studies is through computed tomography (CT) scan or colonoscopy. Here, we are presenting the first case report of surgery specimen assisted diagnosis of this uncommon condition.

## Case presentation

A 63-year-old Chinese male underwent a laparoscopic radical right hemicolectomy for an adenocarcinoma of the ascending colon (pT4N0M0, Stage II) in June 2020. The CT scan and colonoscopy before surgery were shown in (Figure S1A-E). Pathology showed an invasive, moderately differentiated adenocarcinoma with infiltration beyond serosal tissue. There were no lymphovascular and perineural invasion and no lymph node metastasis out of seventeen lymph nodes harvested. Lynch syndrome screen by immunohistochemistry (MLH1, MSH2, MSH6, and PMS2 proteins) showed normal expression in pathological tissue. There were no reportable alterations in KRAS, NRAS, and BRAF. The patient was considered as high risk stage II colon cancer, and therefore, adjuvant chemotherapy with XELOX regimen was started 4 weeks after surgery. The XELOX regimen consisted of a 2-h intravenous infusion of oxaliplatin 130 mg/m^2^ on day 1 and oral capecitabine (trade name: Xeloda) 1000 mg/m^2^ twice daily given for 14 days of a 3-week cycle [1]. On day 2 after finishing capecitabine administration, the patient presented with skin rash, diarrhea, abdominal pain, and vomiting. The symptoms were abated following treatment with dexamethasone, loperamide, and antispasmodic. The following day, the patient developed small amounts of bloody stools 4–5 times per day with occasional fever and abdominal pain. After supportive therapy (antidiarrheal therapy, broad-spectrum antibiotics, and dietary modifications) for 10 days, the bloody stools persist.

**Fig. 1:**
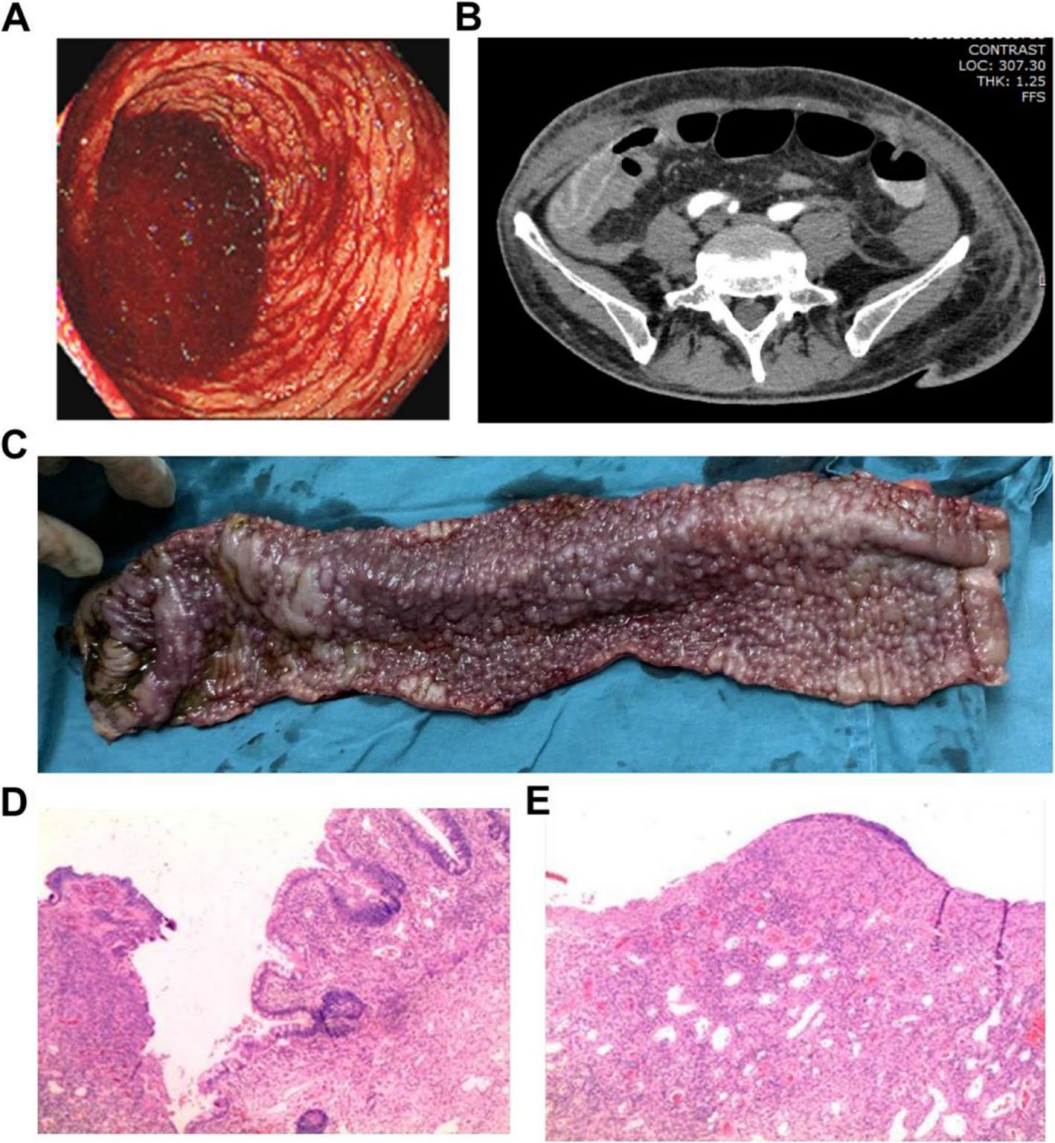
**A** Colonoscopy shows large amount of bloody fluid at the end of the ileum; active bleeding points are difficult to be observed. **B** Computed tomography scan. Axial view showing submucosal edema involving the terminal ileum, with surrounding fat stranding, consistent with ileitis. Mucosal enhancement was preserved. **C** Surgery specimen revealing diffuse inflammatory changes with cobble-like signs and scattered superficial ulcerations on the surface of the terminal ileum. **D** Pathological examination shows diffuse ulceration and erosions in the mucosa with moderate eosinophilic and neutrophil infiltrates

He presented to the gastrointestinal surgery department with anemic appearance, fatigue, and bloody diarrhea. The patient was a nonsmoker and drank alcohol occasionally. There was no relevant medication history or family history. His abdomen was not distended. There was no tenderness or rebound tenderness upon palpation. Bowel sounds were hyperactive. After admission, the patient excreted about 400 ml of crimson colored stools on 2 occasions. The patient developed shock, with a blood pressure of 65/35 mmHg, a temperature of 38.3 °C, tachycardia at 106 beats/min, and hemoglobin of 7.6 g/dl. His white cell count (WCC) was 4.5 × 109/L (normal 3.5–9.0 × 109/L), neutrophils were 2.3 × 109/L (normal 1.5–8 × 109/L) (Figure S1F), and the serum albumin was 19.8 g/L. Stool culture for *Clostridium difficile* (*C. difficile*) was negative. The patient was treated with fluid replacement, blood transfusion, octreotide, losec, hemostatic, and antibiotics. The patient’s condition was stabilized, and a digital subtraction angiography (DSA) (Figure S2D-F) was performed. However, no obvious signs of arterial bleeding were found. On day 2 after admission, the patient again excreted 400 ml of crimson colored stools. An emergency colonoscopy was conducted with no obvious abnormalities observed in the colon or anastomosis except a large amount of bloody fluid at the end of ileum indicating the possibility of small bowel hemorrhage (Fig. 1A, S2B, C). Abdomen and pelvis CT with contrast revealed edema and thickening of the distal ileum wall (Fig. 1B, S2A).

Because of the persistent bloody diarrhea and negative findings of active bleeding points by DSA and colonoscopy, an emergency laparotomy was performed. No diverticulum, arteriovenous malformation, or ischemic necrosis was observed in the whole small intestine and colon except a dilation and thickening in about 40 cm of the terminal ileum. Intraoperative colonoscopy found diffuse inflammatory changes in about 30 cm long of terminal ileum with cobble-like signs and scattered superficial ulcerations as well as bleeding points on the ulcer surface (Fig. 1C). The abnormal segment of ileum including a 5-cm proximal segment and including the previous ileo-colic anastomosis was removed. A new ileo-colic end-to-end manual anastomosis was performed. The pathological examination of the surgery specimen showed an active stage of chronic inflammatory changes. Diffuse ulceration and erosions in the mucosa with moderate eosinophilic and neutrophil infiltrates were observed. Signs of pseudomembrane formation and necrosis were absent (Fig. 1D, E). The bloody diarrhea resolved after surgery and stools were improved in frequency. Given the severity of his life-threatening symptoms, the patient decided not to receive additional adjuvant chemotherapy. As of this writing, 1 year has passed from the ileal resection, and fortunately, the patient has no signs of cancer relapse. The authors are closely monitoring the progress of the patient and preparing to give further treatment advice as needed.

Written informed consent for publication of clinical details and clinical images were obtained from the patient.

## Discussion and conclusions

Gastrointestinal bleeding from adjuvant chemotherapy is rare, and reports of acute severe small bowel bleeding are even rarer following adjuvant XELOX treatment. It can be life-threatening if the bleeding is not promptly diagnosed and treated. Injury of the intestinal mucosa and subsequent enterocolitis induced by chemotherapy appeared to be the main contributing factor for the bleeding. A search of PubMed found only 11 published cases describing capecitabine treatment-related ileitis, and only 1 of these cases documents bloody diarrhea after capecitabine administration [5]. We are presenting the first case report of surgery specimen assisted diagnosis of this condition in combination with colonoscopy and CT scan (Table 1). Our analysis, together with previous literature [5–12], demonstrates that mucosal erosion and ulceration with acute as well as chronic inflammation are commonly seen in capecitabine associated ileitis. Another chemotherapeutic agent in the XELOX regimen is oxaliplatin, and bloody diarrhea caused by this drug is also rare. In a retrospective study of 36,595 patients who received platinum-based therapy, only 37 cases presented blood or mucous in the stools. In these cases, platinum-related colitis and ulcer were found to be the etiology of the hemorrhage [13].

**Table 1:** Previous case reports of capecitabine associated ileitis

Case	Patients information	Chief complaints	Bleeding	Diagnostic findings
Case 1 Barton 2006 [6]	54-year-old man Adenocarcinoma of the right colon Adjuvant chemotherapy: capecitabine	>C3: severe cramps, diarrhea	No	Colonoscopy with biopsy: ulcerative ileitis with eosinophilic infiltrates
Case2 Bouma 2011 [7]	73-year-old man Rectal cancer with liver metastasis Palliative chemotherapy: bevacizumab + oxaliplatin + capecitabine	Abdominal pain, nausea, diarrhea, and subfebrile temperature	No	CT scan: bowel wall thickening particularly of the ileum
Case 3 Al-Gahmi 2012 [8]	65-year-old man Metastatic adenocarcinoma of the rectum Pelvic radiotherapy + XELOX	>C1: fever, abdominal pain, vomiting, and diarrhea	No	Colonoscopy with biopsy: isolated ulceration in the terminal ileum with eosinophilic infiltrates
Case 4 Radwan 2012 [9]	67-year-old man Colon adenocarcinoma Adjuvant chemotherapy: capecitabine	>C2: reduced appetite, lower abdominal discomfort and diarrhea	No	CT scan: thickened edematous distal loops of ileum
Case 5 Mokrim 2014 [10]	66-year-old woman Metastatic breast cancer Palliative chemotherapy: capecitabine	>C1: fever, diarrhea, fatigue, and emesis	No	Colonoscopy with biopsy: ileitis with eosinophilic infiltrates, absence of intraepithelial lymphocytic infiltrates
Case 6 Mokrim 2014 [10]	67-year-old woman Metastatic breast cancer Second-line palliative setting: capecitabine	>C2: diarrhea, fever, fatigue, and reduced appetite	No	CT scan: parietal thickening of the terminal ileal loop
Case 7 Lee 2015 [11]	61-year-old woman Adenocarcinoma of the right colon Second-line adjuvant chemotherapy: capecitabine + irinotecan + cetuximab	>C4: abdominal pain, watery diarrhea, vomiting, and fever	No	CT scan: extensive submucosal edema at the terminal and middle part of the ileum
Case 8 Lee 2015 [11]	59-year-old woman Adenocarcinoma of the sigmoid colon Adjuvant chemotherapy: capecitabine	>C3: diarrhea, hand-foot-skin reaction, and stomatitis	No	CT scan: diffuse submucosal edema with multiple small gas bubbles along the distal ileum
Case 9 Van Hellemond 2018 [12]	69-year-old woman Adenocarcinoma of the sigmoid colon Adjuvant chemotherapy: XELOX	>C1: diarrhea, nausea, and reduced appetite	No	Colonoscopy with biopsy: ileitis with superficial but extensive ulceration in the terminal ileum
Case 10 Dao 2019 [5]	72-year-old woman Adenocarcinoma of the right colon Adjuvant chemotherapy: capecitabine	Watery diarrhea	No	Colonoscopy with biopsy: granular erythematous mucosa erosion with acute inflammation and occasional atypical glands in the terminal ileum
Case 11 Dao 2019 [5]	42-year-old woman Recurrent right breast cancer Palliative chemotherapy: capecitabine	Voluminous bloody diarrhea, abdominal pain, and fever	yes	Colonoscopy with biopsy: diffuse pseudomembranes with inflammatory exudates and spontaneous bleeding in the terminal ileum
Our case	72-year-old woman Adenocarcinoma of the right colon Adjuvant chemotherapy: XELOX	>C1: Voluminous bloody diarrhea, abdominal pain, and fever	yes	Surgery with biopsy: active stage of chronic inflammatory changes: diffuse ulceration and erosions in the mucosa with eosinophilic and neutrophil infiltrates

>C: symptoms onset after cycles of chemotherapy

Pseudomembranous enterocolitis, ischemic enterocolitis, neutropenic enterocolitis, and eosinophilic colitis have been reported as the main types of enteritis that occur in patients presenting with bloody stools after chemotherapy [14]. Yokoyama et al. reported three patients presenting with bloody diarrhea secondary to pseudomembranous colitis (PMC) during or after 5-FU or platinum-based chemotherapy [15]. *C. difficile* and its toxins are considered the main cause of PMC [15]. Intestinal mucosal injury together with neutropenia and the immunocompromised state of the afflicted patients are the main elements in neutropenic colitis (NE). Chemotherapeutic agent toxicities, intestinal leukemic infiltration, and superimposed infections are possible mechanisms in the pathogenesis of NE [16]. Symptoms often appear within 2 weeks following the completion of chemotherapy and coincide with the low leukocyte count following chemotherapy [17]. Patients with neutrophil counts < 500/μL are at increased risk for developing NE [18]. Melena or hematochezia are generally less common forms of presentation [19]. Ischemic colitis is another type of colitis which may manifest as bloody stools and intestinal necrosis in patients administrated platinum or gemcitabine-containing regimens [20]. Pathology evaluation plays an important role in clarifying the etiology of chemotherapy-related enterocolitis. In addition, *C. difficile* infection in stool samples and white blood cell counts could also help with differential diagnoses for enterocolitis. In our case, the pathological examination of surgery specimens showed cobblestone like changes and superficial ulcerations on the surface of terminal ileum; signs of pseudomembrane formation and necrosis were not found. Neutropenia was not observed all through the first cycle of XELOX in this patient, so neutropenic enterocolitis was also ruled out. Although rare, 5-FU-induced allergic and inflammatory colitis was reported by Cappell et al. [21]. The presence of skin rash may suggest a drug allergy in this case. However, eosinophilic infiltrates were not found in the mucosa and submucosa after detailed pathologic examination, indicating allergic enterocolitis [22] was not present. A drug-induced lymphocyte stimulation test (DLST) for 5-FU could be applied to diagnose allergy and provide evidence of allergic enterocolitis. However, its limited sensitivity needs to be noticed when interpreting the results [23]. Patients with inflammatory bowel disease (IBD) could also present with bloody diarrhea. Endoscopy and abdominal CT scan are important methods to establish this diagnosis [24, 25]. In our case, colonoscopy with intubation of the terminal ileum prior to the right hemicolectomy showed no signs of erythema, loss of normal vascular pattern, granularity, erosions, bleeding, or ulcerations except in relation to the neoplasm located in the ascending colon (Figure S1 B-E). Abdominal contrast CT scan before the first surgery showed no evidence of intestinal wall thickening or comb sign (Figure S1A). Together with the timing of the first bloody stools occurring 2 days after finishing capecitabine chemotherapy, the authors felt IBD could be safely excluded. Another chemotherapeutic agent in the XELOX regimen is oxaliplatin, which also should be considered as the etiology of the hemorrhage. It has been reported that the symptoms of platinum-induced colitis develop at a median of 66 days after platinum chemotherapy [13]. In this case, the patient presented to his surgeon with skin rash, fever, and severe bloody diarrhea 2 days after the end of the 2-week capecitabine administration cycle, and the bloody diarrhea persisted until laparotomy. Based on this timing, the authors suspect capecitabine was the main contributor to the hemorrhage, not oxaliplatin. Of note, oxaliplatin-induced immune-mediated thrombocytopenia may also cause life-threatening bleeding. However, the early onset of symptomatic grade 3–4 thrombocytopenia and rapid resolving process after discontinuation of oxaliplatin may help us to differentiate this possibility [26]. Although the cause of ileitis and subsequent bleeding in our patient could not be definitively identified, the evidence presented implicates toxicity of capecitabine on the intestinal mucosa as the most likely mechanism.

It has been well-recognized that dihydropyrimidine dehydrogenase (DPD) is the key enzyme required for 5-FU metabolism. The deficiency of DPD leads to acute severe toxicity after administration of 5-FU or capecitabine [10]. Regulatory agencies in China have not yet taken steps toward systematic pharmacogenetic testing with 5-FU, and neither has our cancer center. This is probably because of the controversial genotype-to-phenotype relationships described in DPD along with the lack of consensus as to a reference phenotype-based method [27]. In addition, clinical reports based solely on the search for DPD genetic mutations largely underestimate the actual effect of DPD deficiency in the severe toxicities encountered with 5-FU [28]. DPD deficiency is obviously not the sole determinant of 5-FU toxicity [29], and clinical decisions based solely on the screening of DPD deficiency might therefore be misleading. Consequently, no regulatory step has been undertaken yet to recommend DPD testing as part of routine clinical practice for securing the administration of 5-FU. Owing to the wide range of phenotype-based techniques presently available in addition to specific genotype screening, the amount of clinical evidence warranting preliminary screening for DPD status as a way to ensure better safety in administering 5-FU is increasing recently [30, 31]. Our life-threatening capecitabine-related toxicity case provides another stark reminder that integration of systematic testing for DPD as part of routine clinical practice may be ideal. Unfortunately, in our case, the consent of testing DPD status was not obtained from the patient afterwards because he decided not to receive additional adjuvant therapy after this terrible life-threatening event. We are closely monitoring this patient for relapse and are prepared to give advice for further treatment if needed. In addition, it has been reported that right colectomy, advanced age, and concurrent use of angiotensin II receptor blockers were associated with adjuvant XELOX-induced severe enterocolitis and rectal bleeding [32]. Awareness of these risk factors allows us to improve patient selection for chemotherapy.

In this case, if continuation of treatment is desired, patient and tumor characteristics should be taken into account. In a palliative setting, toxicity is an important factor influencing quality of life. Therefore, resuming capecitabine in a palliative setting would be inappropriate if the patient experienced capecitabine-induced severe terminal ileitis. In an adjuvant setting, the absolute risk reduction of relapse has to be weighted against the risk of toxicity. Hence, a re-challenge with capecitabine dose reduction or use of an alternate chemotherapy agent might be a valid option. A recent Japanese study showed that an initial administration of a small amount of capecitabine with a gradual dose increase can eventually increase the DPD protein level up to 12-fold before chemotherapy in patients with DPD deficiency [33]. This new way of drug administration is worthy of exploration in future clinical practice. In addition, other oral fluoropyrimidine-based agents with lower toxicities such as S1 [34] or trifluridine-tipiracil (TAS-102) [12, 35–37] could be alternative treatment options. Targeted therapy as well as immunotherapy could also be given after MDT (multidisciplinary diagnosis and treatment) discussion.

The location of gastrointestinal bleeding after chemotherapy may span from the esophageal to the rectum [38]. Upper and lower endoscopy plays an important role in diagnosis and hemostasis in patients refractory to conservative interventions. However, the efficacy of colonoscopy or gastroscopy in the diagnosis and treatment of ileal or jejunal bleeding is limited. Small bowel enteritis and subsequent bleeding post-chemotherapy could be underestimated or misdiagnosed because of this limitation. Using CT scans, Kuebler et al. found that bowel injury occurs more frequently in the small bowel compared to the colon after administering the FLOX regimen (weekly bolus 5-fluorouracil and leucovorin for 6 weeks plus oxaliplatin on weeks 1, 3, and 5 of each cycle). In addition, the involvement of the ileum was more often noted than other sites within the small bowel [39]. Felismino et al. also reported that the most frequent segment affected was the ileum in patients treated with adjuvant XELOX chemotherapy [28]. Hence, small bowel hemorrhage (especially from the ileum) could be considered if DSA and endoscopy yield negative results. Once life-threatening hemodynamic instability occurs, an emergency laparotomy should be performed if endoscopy and supportive treatments are ineffective.

In conclusion, gastrointestinal bleeding following capecitabine treatment in colorectal cancer patients might be life-threatening. Injury of the intestinal mucosa and subsequent ileitis induced by capecitabine should always be considered in the differential diagnosis of severe gastrointestinal bleeding. Awareness of the risk factors such as deficiency of DPD, advanced age, or right colectomy allows us to make more individualized chemotherapy decisions. When severe bleeding occurs, emergency surgery may achieve satisfactory outcomes if medical and endoscopic interventions are ineffective. The application of genetic screens in identifying patients who are likely to develop capecitabine toxicity is promising and may aid in reducing morbidity in the future. Restarting capecitabine from a small amount with a gradual dose increase might be attempted in patients with DPD deficiency, although further research is required to validate safety outcomes.

## Supplementary Information

**Additional file 1: Figure S1. A.** Computed tomography scan. Axial view shows an obvious colonic wall thickening at the ascending colon. **B-E.** Colonoscopy shows a neoplasm located at the ascending colon with slight lumen stenosis. **(B)**. No signs of erythema, loss of normal vascular pattern, granularity, erosions bleeding and ulcerations across the rest of bowel lumen. (**C-E**, **C**. terminal ileum, **D**. transverse colon, **E**. descending colon) **F.** Run chart of neutrophil counts all through this clinical treatment course. **Figure S2. A.** Computed tomography scan. Coronal view showing submucosal edema involving the terminal ileum. Mucosal enhancement was preserved. **B, C**. Colonoscopy shows no obvious abnormalities including ulceration or bleeding in the remaining colon **(C)** and anastomosis **(B)**. **D-F.** Negative findings of active bleeding points by digital substraction angiography (DSA).

## Data Availability

Data are available on request from the authors.
