# Supplementary material for: Severe ileum bleeding following adjuvant capecitabine chemotherapy for locally advanced colon cancer: a case report and review of the literature
Source: World J Surg Oncol. 2021 Nov 22;19:332. doi: 10.1186/s12957-021-02443-8 (PMC8609886; doi:10.1186/s12957-021-02443-8)

Figure S1

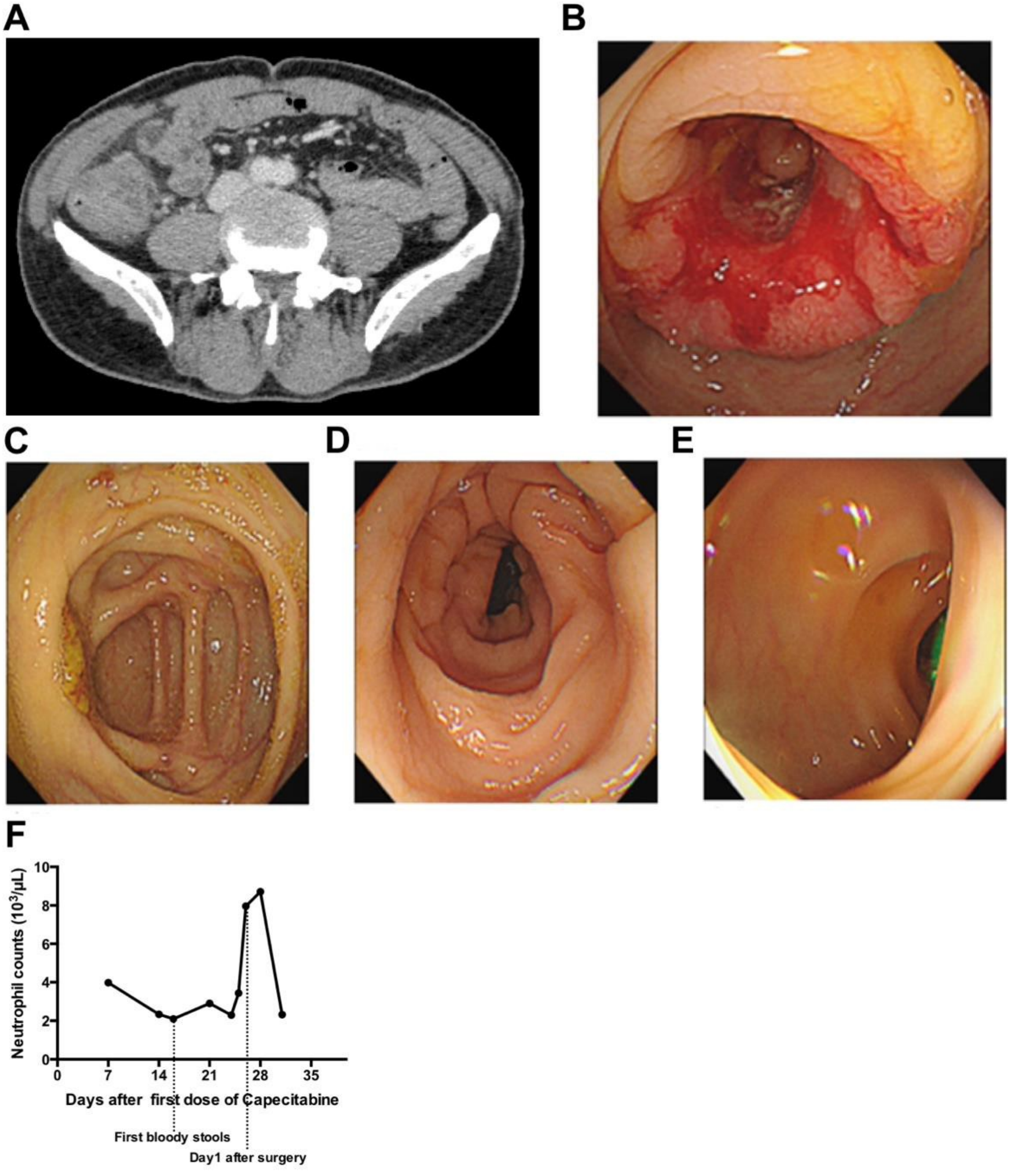

A
B
C
D
E
F
Neutrophil counts (10³/μL)
10
8
6
4
2
0
0
7
14
21
28
35
Days after first dose of Capecitabine
First bloody stools
Day1 after surgery

Figure S2

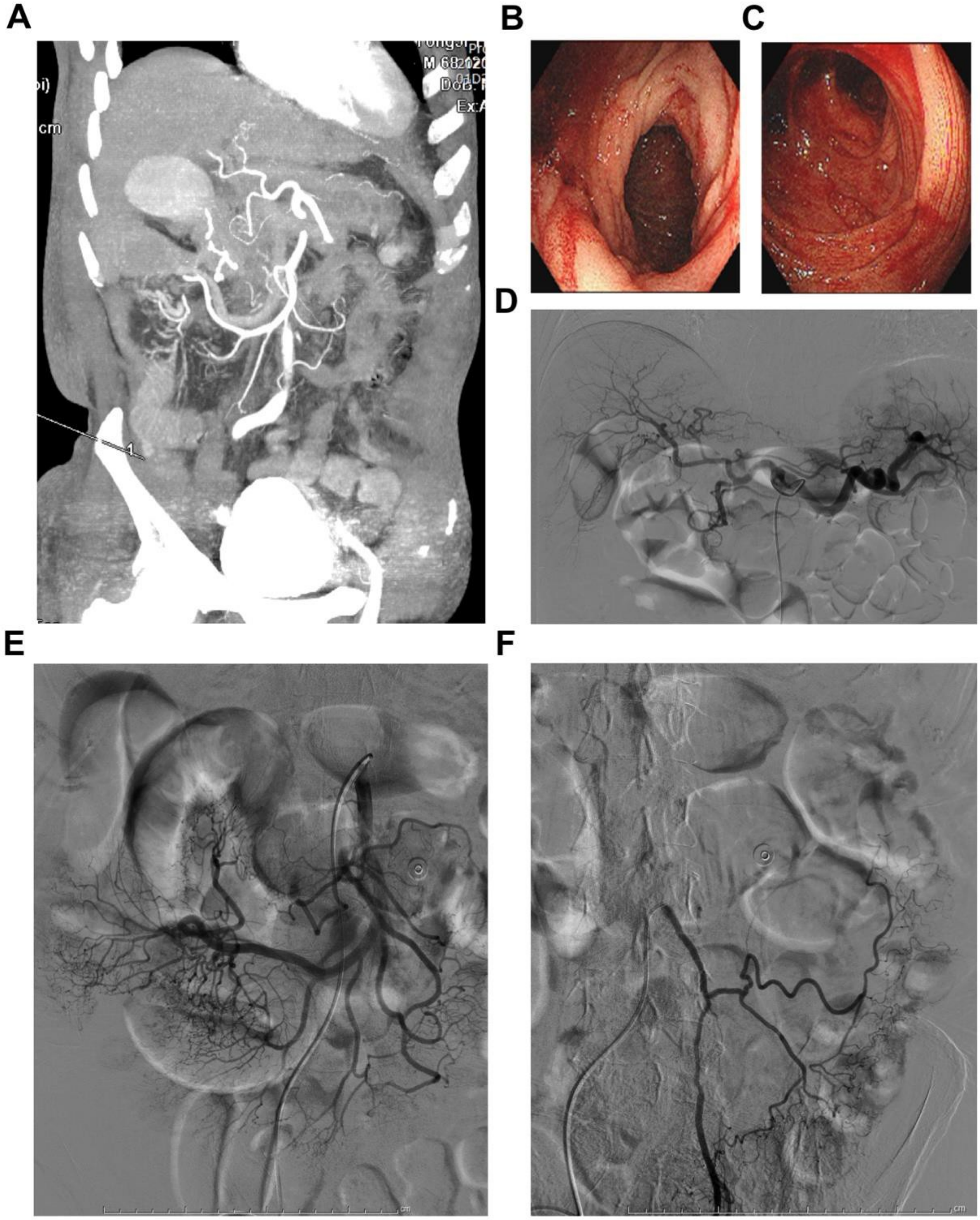

Supplement: Supplementary file 1 [file 12957_2021_2443_MOESM1_ESM.docx]
